# Interplay between Ubiquitin, SUMO, and Poly(ADP-Ribose) in the Cellular Response to Genotoxic Stress

**DOI:** 10.3389/fgene.2016.00063

**Published:** 2016-04-19

**Authors:** Stefania Pellegrino, Matthias Altmeyer

**Affiliations:** Department of Molecular Mechanisms of Disease, University of ZurichZürich, Switzerland

**Keywords:** ubiquitin, SUMO, poly(ADP-ribose), PARP, DNA damage response, DDR, genome stability, cancer

## Abstract

Cells employ a complex network of molecular pathways to cope with endogenous and exogenous genotoxic stress. This multilayered response ensures that genomic lesions are efficiently detected and faithfully repaired in order to safeguard genome integrity. The molecular choreography at sites of DNA damage relies heavily on post-translational modifications (PTMs). Protein modifications with ubiquitin and the small ubiquitin-like modifier SUMO have recently emerged as important regulatory means to coordinate DNA damage signaling and repair. Both ubiquitylation and SUMOylation can lead to extensive chain-like protein modifications, a feature that is shared with yet another DNA damage-induced PTM, the modification of proteins with poly(ADP-ribose) (PAR). Chains of ubiquitin, SUMO, and PAR all contribute to the multi-protein assemblies found at sites of DNA damage and regulate their spatio-temporal dynamics. Here, we review recent advancements in our understanding of how ubiquitin, SUMO, and PAR coordinate the DNA damage response and highlight emerging examples of an intricate interplay between these chain-like modifications during the cellular response to genotoxic stress.

## Introduction

Our genetic material is under constant cellular surveillance and care. Maintaining genome stability is indeed a vital task, not only under conditions when external toxins or physical strains challenge the integrity of the genome, but also in the course of normal cellular metabolism, when reactive metabolites and physiological DNA transactions can lead to a plethora of lesions. If these damages are not detected and faithfully repaired, cells run the risk of accumulating mutations that can erode genome function, vitiate cell fate, or compromise cell survival. Faced with such threats cells have developed sophisticated mechanisms to sense and repair damaged DNA. These mechanisms, which are collectively referred to as the DNA damage response (DDR), not only ensure that most lesions are efficiently repaired, but they also coordinate genome integrity maintenance with other cellular functions such as transcription, DNA replication, and cell cycle progression (Ciccia and Elledge, 2010). The DDR is an intricate molecular network that safeguards genome integrity and helps to maintain cell identity, thus constituting a natural barrier against the development of various human diseases (Jackson and Bartek, 2009). Underpinning the crucial role of genome integrity maintenance for human health, a deteriorated DDR and signs of genome instability are typical features of many human cancers, and they represent cancer-specific vulnerabilities that can be targeted by precision therapies (O’Connor, 2015).

To fulfill its task, the DDR employs a multitude of tightly regulated posttranslational protein modifications (PTMs). In addition to modulating protein functions locally at the damage site, PTMs play important roles in spreading the DNA damage signal to the surrounding chromatin (Lukas et al., 2011; Polo and Jackson, 2011) and in activating cell cycle checkpoints (Stracker et al., 2009). Positive feedback mechanisms amplify the DNA damage signal and enable sustained accumulation of genome caretaker proteins, while antagonistic mechanisms ensure that modifications induced by DNA damage remain spatially and temporally confined (van Attikum and Gasser, 2009; Altmeyer and Lukas, 2013a,b; Panier and Durocher, 2013). Multiple PTMs cooperate in this spatio-temporal regulation and can either act in series, in parallel or in a combinatorial fashion to dynamically reshape the chromatin landscape around DNA lesions and prepare the stage for repair (Dantuma and van Attikum, 2016). This barcoding involves multi-target phosphorylation (Marechal and Zou, 2013; Shiloh and Ziv, 2013; Davis et al., 2014; Awasthi et al., 2015; Paull, 2015), as well as acetylation and methylation (Gong and Miller, 2013; Hendzel and Greenberg, 2013; Price and D’Andrea, 2013). In addition to these small moiety modifications, recent work revealed how larger PTMs, which can form extensive modification chains, coordinate the access of genome caretakers to DNA lesions and regulate repair pathway choices. Here, we briefly discuss how ubiquitylation, SUMOylation, and poly(ADP-ribosyl)ation (PARylation) are employed by the DDR, before we highlight emerging examples that have started to elucidate an intricate and still incompletely understood crosstalk between these catenarian modifications in response to DNA damage. We focus our analysis primarily on the response of mammalian cells to DNA double strand breaks (DSBs), yet an equally well-coordinated crosstalk between chromatin-based PTMs also operates in other situations of genotoxic stress (Kim and D’Andrea, 2012; Marteijn et al., 2014; Ulrich, 2014).

## Ubiquitin Conjugation Around DSB Sites

**FIGURE 1:**
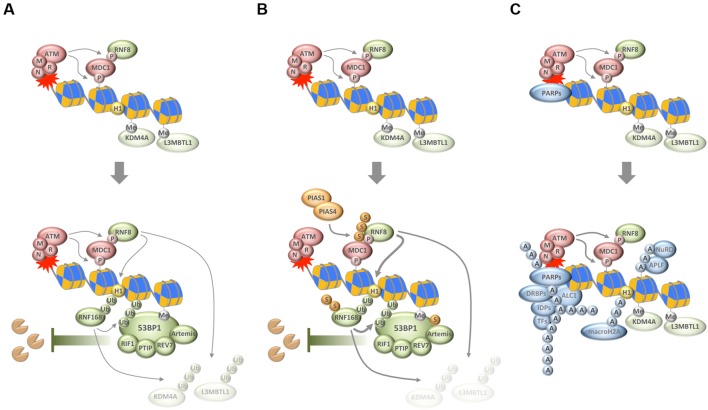
**Chain-like modifications build up dynamic DNA repair compartments that orchestrate the DNA damage response (DDR). (A)** In response to DNA damage, and subsequent to the MRN/ATM/MDC1-driven phosphorylation of histone variant H2AX, ubiquitylation of H1 and H2B by RNF8 and RNF168, respectively, synergizes with ubiquitin-dependent extraction of proteins from the damaged chromatin to promote the recruitment of 53BP1 and its downstream effectors. **(B)** SUMOylation by PIAS1 and PIAS4 further enhances ubiquitin conjugation around DNA break sites. **(C)** Poly(ADP-ribosyl)ation (PARylation) by PARPs generates a recruitment platform for a plethora of PAR-binding proteins, including various transcription factors (TFs), DNA- and RNA-binding proteins (DRBPs), and a set of intrinsically disordered proteins (IDPs). P, phosphorylation; Me, methylation; Ub, ubiquitylation; S, SUMOylation; A, ADP-ribosylation; M, MRE11; R, RAD50; N, NBS1.

Chromosome breaks are among the most toxic DNA lesions and two major repair pathways evolved to deal with DSBs. The non-homologous end-joining (NHEJ) pathway is independent of intact template DNA sequences and can re-ligate broken DNA ends throughout the cell cycle. In contrast, faithful repair by homologous recombination (HR) depends on an undamaged template DNA and is thus restricted to the S/G2 phases of the cell cycle when sister chromatids are available. While NHEJ is generally considered error-prone due to the risk of nucleotide loss from DNA ends, HR is considered to be more accurate due to template-based repair. The choice between NHEJ and HR is tightly controlled, and imbalances in its regulation can lead to genome instability and accelerate cancer development (Chapman et al., 2012; Aparicio et al., 2014). Interestingly, the recruitment of several key repair pathway choice mediators to DNA break sites depends on local ubiquitin conjugations (Messick and Greenberg, 2009; Pinder et al., 2013). Indeed, one of the central players of repair pathway choice is the ubiquitin-sensing genome caretaker protein 53BP1, whose recruitment to DSBs requires the consecutive action of the ubiquitin E3 ligases RNF8 and RNF168 (Panier and Boulton, 2014). In a concerted manner, and initiated by upstream phosphorylation of the histone variant H2AX, RNF8, and RNF168 ubiquitylate histones H1 and H2A, respectively, and thereby provide a landing platform for 53BP1 (Mattiroli et al., 2012; Fradet-Turcotte et al., 2013; Gatti et al., 2015; Thorslund et al., 2015). 53BP1 in turn assembles the effector proteins RIF1, PTIP, Artemis, and MAD2L2/REV7 to limit the extent of DNA end resection and thereby channel repair toward NHEJ (**Figure 1A**) (Callen et al., 2013; Chapman et al., 2013; Di Virgilio et al., 2013; Escribano-Diaz et al., 2013; Zimmermann et al., 2013; Wang et al., 2014; Boersma et al., 2015; Xu et al., 2015). Of note, the functions of 53BP1 and its effectors are required for the hypersensitivity of HR-defective cancer cells to inhibitors of PAR polymerases (Lord and Ashworth, 2016), thus linking the consequences of compromised PARylation to the effects of a ubiquitin-dependent anti-resection barrier under pathological repair pathway choice conditions.

Importantly, ubiquitin conjugation is not only involved in generating the ubiquitin code that is recognized by 53BP1, but also fosters 53BP1 accumulation by RNF8/RNF168-dependent and VCP/p97-mediated removal of proteins from damaged chromatin (Acs et al., 2011; Meerang et al., 2011). As was shown for the H4K20me2-binding proteins L3MBTL1 and JMJD2A/KDM4A, this can unmask additional binding sites for the tandem tudor domain of 53BP1 (Acs et al., 2011; Mallette et al., 2012). Thus, the removal of chromatin binders seems to converge with the generation of new chromatin marks to allow for the efficient recruitment of 53BP1 and its downstream effectors (**Figure 1A**).

While these reactions build up an important anti-resection barrier that shields broken DNA ends from unscheduled nucleolytic digestion, ubiquitin conjugation also plays a role in promoting HR. For example, the ubiquitin E3 ligases TRIP12 and UBR5 cooperate to keep RNF168 levels in check and thereby prevent excessive 53BP1 function (Gudjonsson et al., 2012). More recently, the ubiquitin E3 ligase RNF138 was shown to accumulate at sites of DNA damage where it stimulates DNA end resection and promotes HR (Ismail et al., 2015; Schmidt et al., 2015). Thus, rather than channeling repair pathway choices in one direction, the ubiquitylation system employs rheostats and antagonistic sub-pathways to regulate repair decisions in a manner that likely integrates information about cell cycle phase and chromatin context.

## SUMO Conjugation at DSB Sites Assists the DDR

Just like ubiquitylation, also SUMOylation plays important roles for the tightly controlled protein choreography at DSB sites, and its deregulation impairs genome stability and cell proliferation (Bekker-Jensen and Mailand, 2011; Jackson and Durocher, 2013; Eifler and Vertegaal, 2015). The SUMO isoforms SUMO1 and SUMO2/3 were all found to accumulate at sites of DNA damage in a manner dependent on the SUMO E3 ligases PIAS1 and PIAS4 (Galanty et al., 2009; Morris et al., 2009). Interestingly, the SUMO conjugation observed in response to DNA breakage promotes accumulation of ubiquitin chains on damaged chromatin and is required for the efficient recruitment of ubiquitin-dependent genome caretakers (Galanty et al., 2009; Morris et al., 2009). Among the targets of DNA damage-induced SUMOylation are MDC1, RNF168, 53BP1, BRCA1, RPA, and EXO1 (**Figure 1B**) (Galanty et al., 2009; Morris et al., 2009; Luo et al., 2012; Yin et al., 2012; Bologna et al., 2015; Hendriks et al., 2015). SUMOylation not only contributes to the recruitment of proteins to DSBs but also to their coordinated removal, and, interestingly, is required for both NHEJ and HR (Galanty et al., 2009; Morris et al., 2009; Chung and Zhao, 2015; Sarangi et al., 2015). While much remains to be learnt about the exact mechanisms how SUMOylation and SUMO chain formation in particular affect the repair of DSBs, it has become clear that the SUMOylation and ubiquitylation machineries work closely together to help restore genome integrity upon chromosome breakage (see below).

## Poly(ADP-Ribose) Chains Attract a Diverse Set of Proteins to DNA Break Sites

A third type of protein modification that comes in chains and ties proteins to DNA breaks sites is PARylation. Catalyzed by PAR polymerases (PARPs) in response to genotoxic stress, DNA break-associated ADP-ribose polymers provide a landing platform for a plethora of PAR-binding proteins (Teloni and Altmeyer, 2016). This includes chromatin remodelers and DNA repair factors, but also proteins involved in nucleic acid metabolism and RNA processing (Krietsch et al., 2013; Golia et al., 2015; Izhar et al., 2015; Teloni and Altmeyer, 2016). PAR-dependent events have been implicated in the cellular response to DNA single-strand breaks and in maintaining the integrity of perturbed replication forks, but also contribute to DSB repair (Beck et al., 2014). Among the proteins that respond to PAR formation are the DDR factors MRE11 and NBS1 (Haince et al., 2008), the chromatin remodeler ALC1 (Ahel et al., 2009; Gottschalk et al., 2009), the histone variant macroH2A (Timinszky et al., 2009), components of the repressive polycomb and NuRD complexes (Chou et al., 2010; Polo et al., 2010), NHEJ and HR factors (Ahel et al., 2008; Rulten et al., 2008; Li and Yu, 2013; Zhang et al., 2015), and a class of intrinsically disordered proteins that can phase separate to generate dynamic compartments (**Figure 1C**) (Altmeyer et al., 2015; Patel et al., 2015). The relative contribution of each of these recruitments for faithful DNA repair is insufficiently understood and may depend on the type of damage and its complexity as well as the overall damage load. The amount and type of damage, together with cell cycle phase and local chromatin environment, are likely to influence the number of PAR chains generated, their length and branching frequency, and may thereby impact on the protein recruitments that are driven by PAR formation.

Given that all three catenarian modifications, ubiquitylation, SUMOylation, and PARylation, orchestrate the protein accumulations around DNA break sites, significant crosstalk exists. In the following paragraphs, we highlight emerging examples of such interplay and how it affects the accrual of genome caretakers at damaged chromatin.

## Interplay between Ubiquitin and SUMO

**FIGURE 2:**
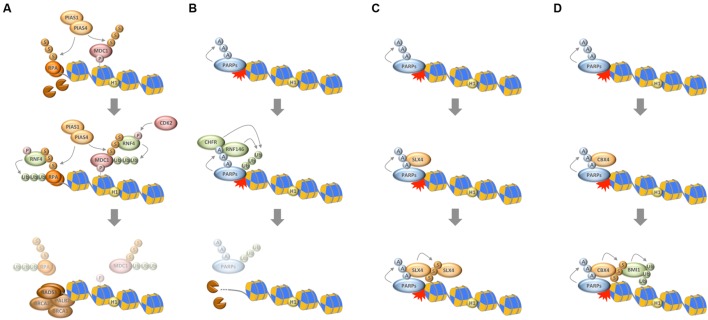
**Interplay between chain-like modifications at sites of DNA damage. (A)** Interplay between ubiquitylation and SUMOylation through the STUbL RNF4, which ubiquitylates SUMOylated substrates to mediate their timely removal from repair sites. **(B)** Interplay between PARylation and ubiquitylation through the PTUbLs RNF146 and CHFR, which cooperate to dissociate automodified PARP1 from DNA break sites. **(C)** Productive interaction between PARylation and SUMOylation to stabilize the recruitment of the SLX4 complex. **(D)** Interplay between PARylation, SUMOylation, and ubiquitylation via the PAR-responsive SUMO ligase CBX4 and the SUMO-responsive ubiquitin ligase BMI1 to promote chromatin ubiquitylation in response to DNA damage. P, phosphorylation; Ub, ubiquitylation; S, SUMOylation; A, ADP-ribosylation.

As noted above, the ubiquitylation and SUMOylation machineries are tightly interconnected and cooperate to reshape the chromatin landscape for proper repair (Bekker-Jensen and Mailand, 2011; Jackson and Durocher, 2013). An interesting direct link between the two systems is provided by SUMO-targeted ubiquitin ligases (STUbLs), readers of SUMO modifications that possess ubiquitin ligase activity and specifically modify SUMOylated substrates. The STUbL RNF4 is a prime example that recently emerged as important regulator of protein accumulation upon DNA breakage. RNF4 is recruited to DSBs via its SUMO interaction motifs and ubiquitylates SUMOylated DDR factors, thereby leading to their withdrawal from repair sites and initiating their proteasomal degradation (Galanty et al., 2012; Luo et al., 2012; Yin et al., 2012). Defective targeting by RNF4 enhances the retention of a subset of DDR factors and compromises the initiation of downstream events required for efficient repair. Among the proteins that are targeted by RNF4 is the adaptor protein MDC1, whose removal promotes access of the DNA end resection and HR machineries (Galanty et al., 2012; Luo et al., 2012; Yin et al., 2012). Once DNA end resection has occurred, RNF4 is again required for the extraction of the single-stranded DNA binding protein RPA, which in turn allows for the accumulation of BRCA2 and RAD51 on resected DNA (Galanty et al., 2012). Collectively, these findings suggest that SUMO-targeted ubiquitylation participates in the dismantling of the anti-resection barrier and promotes HR reactions. In support of this notion, the activity of RNF4 itself is regulated in a CDK-dependent manner, allowing it to fulfill its HR-promoting roles primarily in the S/G2 phases of the cell cycle (**Figure 2A**) (Luo et al., 2015; Kuo et al., 2016). Interestingly, the DNA damage-induced crosstalk between SUMOylation and ubiquitylation is not restricted to DSBs (Poulsen et al., 2013; Ragland et al., 2013; Gibbs-Seymour et al., 2015; van Cuijk et al., 2015), and SUMO-targeted ubiquitylation followed by targeted protein removal and/or degradation thus emerges as a common theme in the stepwise progression of DNA repair pathways.

## Interplay between Poly(ADP-Ribose) and Ubiquitin

In analogy to SUMO-targeted ubiquitylation by STUbLs it was recently discovered that also PAR serves as recognition signal for selected ubiquitin ligases (Zhang et al., 2011; Zhou et al., 2011; Wang et al., 2012). The best-characterized PAR-targeted ubiquitin ligase (PTUbL) is RNF146/Iduna. By virtue of its PAR-binding WWE domain RNF146 is recruited to PARylated proteins where the WWE–PAR interaction leads to an allosteric activation of its ubiquitin ligase domain (DaRosa et al., 2015). Among the proteins that are ubiquitylated by RNF146 are PARP1, PARP2, KU70, DNA ligase III, and XRCC1 (Kang et al., 2011). Thus, and in parallel to DNA break-induced SUMOylation, also PAR participates in the targeted protein ubiquitylation and turnover at sites of genomic lesions.

While these events likely evolved to prevent excessive interactions of repair factors with DNA break sites, PAR-dependent ubiquitylation also assists the early recruitment of genome caretakers. For instance, PAR formation was shown to be required for the recruitment of the BAL1/BBAP ubiquitin ligase complex, whose activity promotes the retention of the RAP80-BRCA1 complex (Yan et al., 2013). This mechanism seems to act in parallel to the PAR-mediated recruitment of BRCA1 via the PAR-binding BRCT domains of its partner protein BARD1 (Li and Yu, 2013), and is part of the PAR-dependent selective interaction filtering that is observed almost immediately upon DNA damage induction and temporally precedes the full build-up of the RNF8/RNF168-dependent ubiquitin compartment (Altmeyer et al., 2015; Teloni and Altmeyer, 2016). Notably, even at later stages of the chromatin response to DNA damage interplay between PARylation and ubiquitylation seems to exist, because the PAR-dependent recruitment of the chromatin remodeler SMARCA5/SNF2H facilitates RNF168 accumulation and promotes efficient ubiquitin conjugation (Smeenk et al., 2013).

Another RING-type ubiquitin E3 ligase, whose role in genome integrity maintenance is linked to PAR formation, is the mitotic regulator CHFR. A PAR-binding zinc finger motif (PBZ) mediates its interaction with genotoxic stress-induced PAR and was shown to be required for the CHFR-dependent antephase checkpoint (Ahel et al., 2008; Oberoi et al., 2010). Interestingly, the functions of CHFR also feed into histone acetylation and ATM activation (Wu et al., 2011), and mediate the first wave of ubiquitylation in response to DNA damage (Liu et al., 2013). As part of this response, CHFR ubiquitylates PARP1 itself, leading to its dissociation from DNA break sites, thus representing another example of PTUbL-mediated stepwise succession of repair events (Kashima et al., 2012; Liu et al., 2013).

Taken together, PARylation assists the early recruitment of genome caretakers, including various ubiquitin ligases, which further promote chromatin modifications and lead to the formation of a dedicated repair compartment, but it also participates in temporarily restraining protein access to repair sites and in the timely and PTUbL-mediated removal of repair factors once they have fulfilled their duties (**Figure 2B**).

## Interplay between Poly(ADP-Ribose) and SUMO

The first direct links between PARylation and SUMOylation were described in the context of PARP1-regulated transcription (Martin et al., 2009; Messner et al., 2009), and SUMOylation of PARP1 was indeed found to be largely irresponsive to DNA damage (Zilio et al., 2013). More recently, however, a functional crosstalk between these two chain-like modifications has started to emerge also in the context of genome integrity maintenance. For instance, PARylation of tyrosyl-DNA phosphodiesterase 1 (TDP1) was shown to cooperate with TDP1 SUMOylation to stabilize the protein and promote its function in the repair of trapped topoisomerase I (TOP1) cleavage complexes (Das et al., 2014). Similarly, PARylation and SUMOylation cooperate to recruit and stabilize the SLX4 nuclease scaffold complex, itself a SUMO E3 ligase, at DNA damage sites (**Figure 2C**) (Gonzalez-Prieto et al., 2015; Guervilly et al., 2015). Finally, and as an example for productive interplay between all three chain-like modifications, PARylation is required for the recruitment of CBX4. In a pathway that functions in parallel to the PIAS1/PIAS4-mediated SUMOylation at damaged chromatin, PAR-dependent SUMOylation by CBX4 attracts the polycomb ubiquitin E3 ligase BMI1, which in turn contributes to DNA damage-induced histone ubiquitylation and promotes repair (**Figure 2D**) (Ismail et al., 2010, 2012, 2015; Ginjala et al., 2011). Thus, intriguing examples of close cooperation between catenarian modifications exist, and future findings are likely to shed more light onto the intricate interplay between ubiquitin, SUMO and PAR in the DDR.

## Conclusion and Outlook

While distinct in their chemical nature and regulatory mechanism, ubiquitylation, SUMOylation, and PARylation share the feature of being chain-like protein modifications. The composition of modification chains, their length, linkage type and branching frequency contains information that can be used by complex regulatory circuits and signaling pathways such as the DDR. Recent work has elucidated how cells employ a sophisticated sequence of reactions with remarkable temporal and spatial resolution to shield genomic lesions and build up dynamic functional platforms that promote repair. The information content imbedded in this response is immense, and the use of modification chains may thus support the need for lesion-specific chromatin barcodes that dynamically change as repair reactions progress.

The multivalent protein recruitment polymers formed by ubiquitylation, SUMOylation, and PARylation often cooperate to achieve robust responses. To this end, they act successively or in parallel, and frequently use positive feedback loops to amplify the signal and increase its specificity. They also employ time-delayed negative feedback to terminate reactions and disassemble complexes, which are no longer needed and constitute roadblocks for downstream events. While recent work has started to elucidate the crosstalk between these modifications, how their combinatorial use and dynamic interplay reshapes the chromatin environment surrounding different types of genomic lesions, dictates repair pathway decisions, and determines repair fidelity remains incompletely understood. Moreover, almost nothing is known about mixed chain modifications, e.g., PARylation of ubiquitin or SUMO chains, and how they might be employed by the DDR. Quantitative time-resolved proteomics and imaging approaches that provide spatial information about protein redistribution and can relate this to cell cycle information are powerful tools to address these issues. The insights gained will not only deepen our understanding of the DDR, but may also provide additional clues to the mechanisms that underlie the toxicity of inhibiting chain-like modifications in cancer treatments.

## Author Contributions

All authors listed, have made substantial, direct and intellectual contribution to the work, and approved it for publication.

## Conflict of Interest Statement

The authors declare that the research was conducted in the absence of any commercial or financial relationships that could be construed as a potential conflict of interest.
